# Supplementary material for: The MinCDE Cell Division System Participates in the Regulation of Type III Secretion System (T3SS) Genes, Bacterial Virulence, and Motility in Xanthomonas oryzae pv. oryzae
Source: Microorganisms. 2022 Jul 31;10(8):1549. doi: 10.3390/microorganisms10081549 (PMC9414521; doi:10.3390/microorganisms10081549)
Supplement: Supplementary file 1 [file microorganisms-10-01549-s001.zip › Supplementary Figures.pdf]

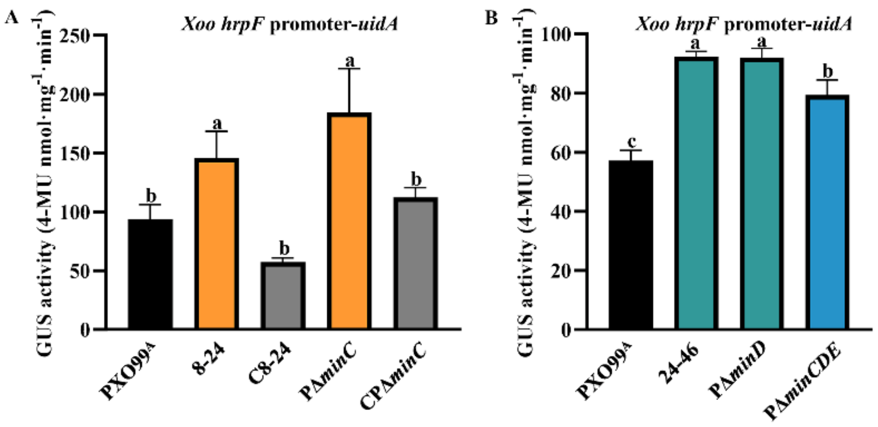

Figure S1. The *Xoo* Min system contributes to *hrpF* gene expression. (A) The *hrpF* promoter-driven GUS activity of PXO99^{A}, 8-24, PΔ*minC*, and the complementary strains C8-24 and CPΔ*minC* measured in XOM3 at 3 h post-induction. (B) The *hrpF* promoter-driven GUS activity of PXO99^{A}, 24-46, PΔ*minD*, and t PΔ*minCDE* measured in XOM3 at 3 h post-induction. Error bars indicate standard deviation. Differences between strains were compared using the LSD test, different letters indicate significant differences ($p < 0.05$).

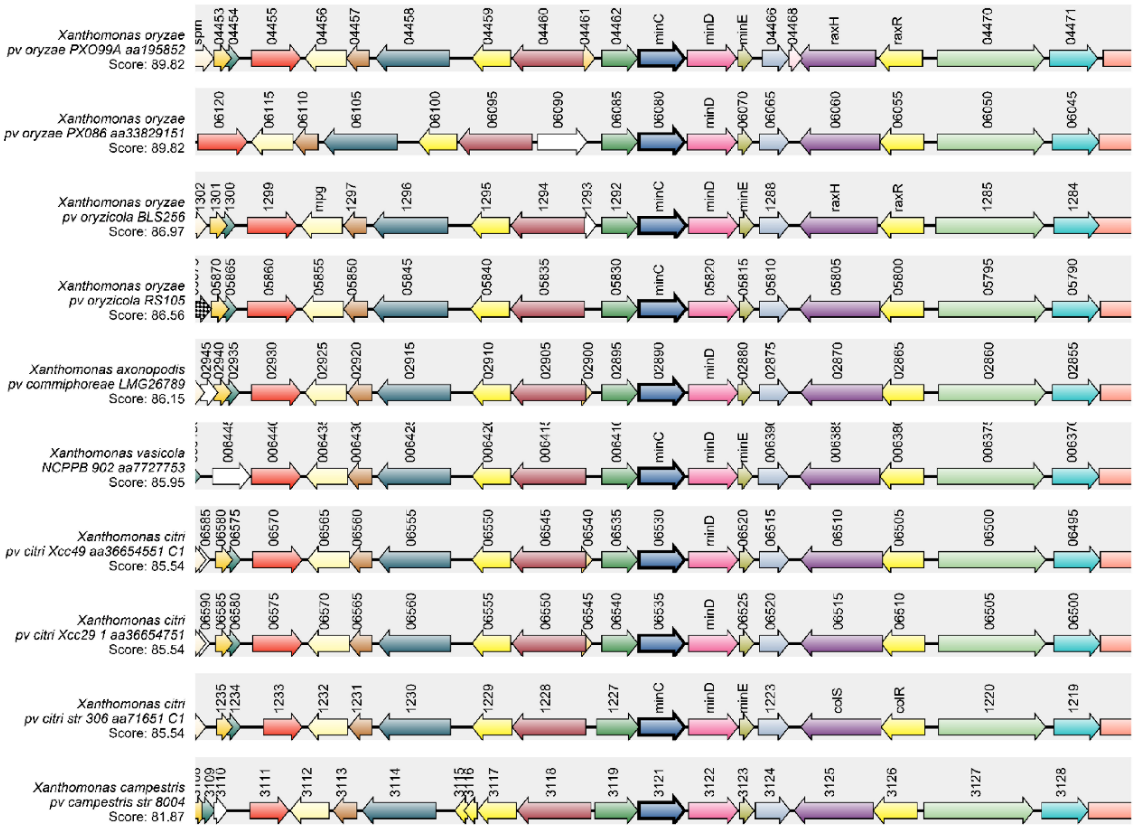

Figure S2. Synteny analysis of the *minCDE* gene cluster among *Xanthomonas* strains. Genomic sections of various *Xanthomonas* spp. were aligned based on the *minC* gene highlighted in black. Synteny analysis was performed using the SyntTax bioinformatics tools provided with the Absynte algorithm (http://archaea.u-psud.fr/synttax/) with ten *Xanthomonas* genomes as a reference. According to the synteny results, the Min system is conserved in *Xanthomonas* species.

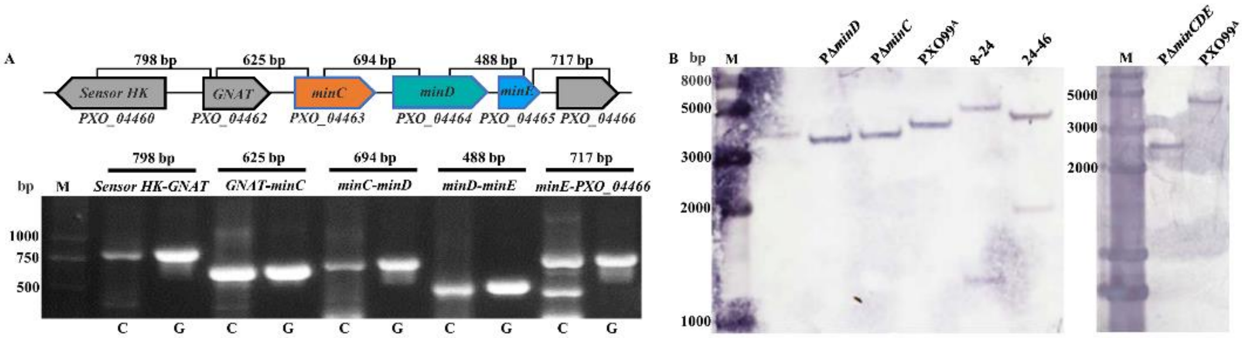

Figure S3. Transcription unit analysis of the *minCDE* gene cluster in *Xoo* PXO99ᴬ and Southern blot analysis of the *Xoo* Min mutants. (A) Co-trascription of *PXO_04462*-minC-*minD*-*minE*-*PXO_04466* by RT-PCR. C: cDNA, G: genomic DNA. (B) Southern blot analysis of the *Xoo* Min mutants PΔ*minC*, PΔ*minD*, PΔ*minCDE*, 8-24 and 24-46. Genomic DNA were digested with *Bam*HI and hybridized with a DNA probe directed against the *minCDE* gene fragment.

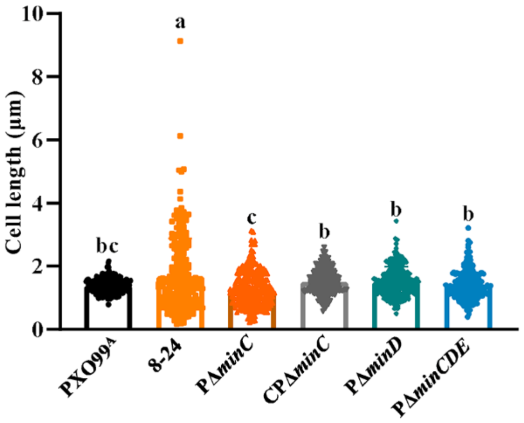

Figure S4. Cell length distribution of the *Xoo* Min mutants 8-24, PΔ*minC*, PΔ*minD*, PΔ*minCDE*, and the complementary strain CPΔ*minC*. Cell lengths of the wild-type PXO99ᴬ, 8-24, PΔ*minC*, CPΔ*minC*, PΔ*minD* and PΔ*minCDE* were calculated by ImageJ software from the pictures of scanning electron microscope (SEM) (n≥200 cells). Differences between strains were compared using the LSD test, different letters indicate significant differences ($p < 0.05$).

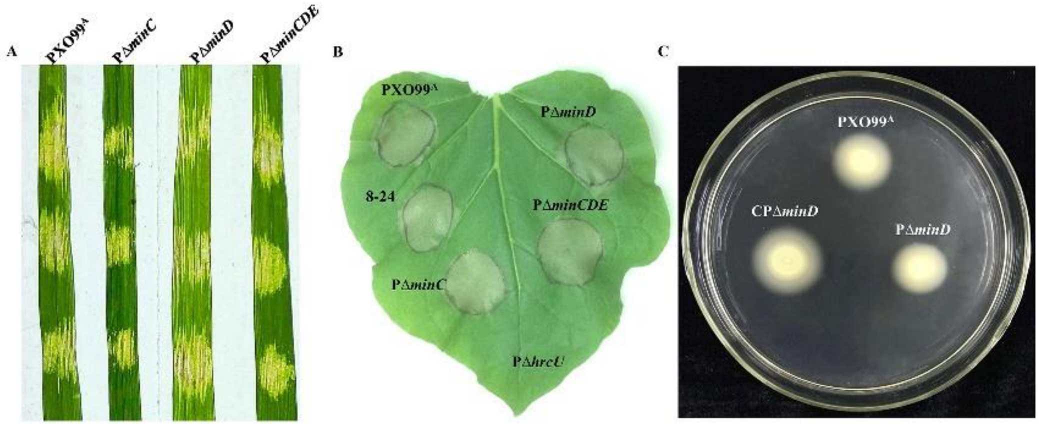

Figure S5. Phenotypes about water-soaked lesions in host rice, hypersensitive response in nonhost tobacco and swimming motility of the Min mutants. (A) Water-soaked lesions on IR24 caused by PXO99^A, PΔ*minC*, PΔ*minD*, and PΔ*minCDE* at 3 days post inoculation. Bacterial suspensions (OD_{600}=1.0) were infiltrated into the leaves of susceptible rice IR24. (B) Hypersensitive response in nonhost tobacco caused by PXO99^A, 8-24, PΔ*minC*, PΔ*minD*, and PΔ*minCDE* at 1 day post inoculation. Bacterial suspensions (OD_{600}=1.0) were infiltrated into the leaves of tobacco. PΔ*hrcU* is a T3SS defective mutant of Xoo PXO99^A as a negative control. (C) Swimming motility of PXO99^A, PΔ*minD*, and CPΔ*minD* on NA medium with 0.15% agar.

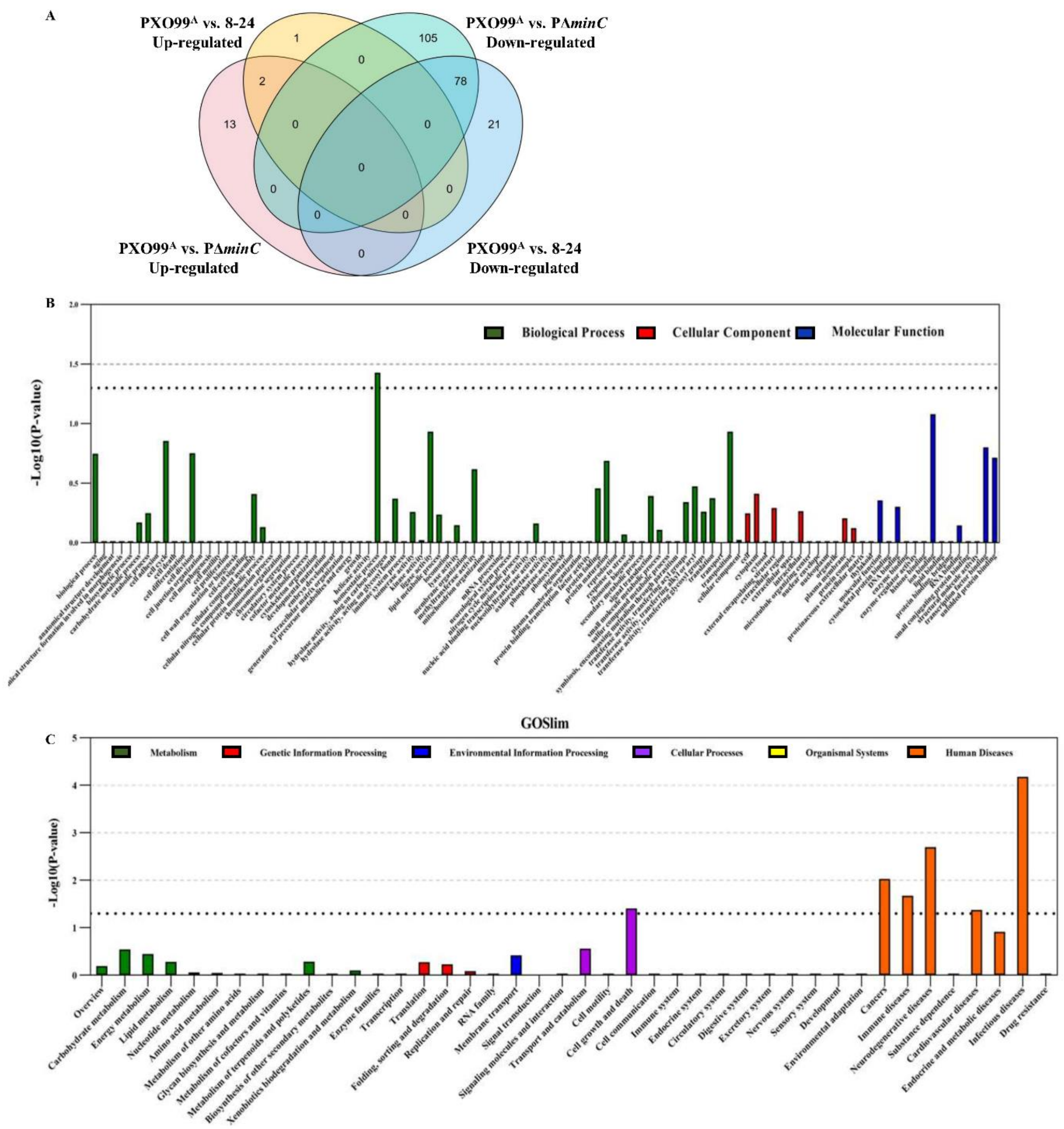

Figure S6. Venn diagram analysis, GO analysis and KEGG analysis of the differentially expressed genes (DEGs) in PXO99^{A} versus in PΔ*minC* and 8-24. (A) **Venn diagram analysis** of the up-regulated and down-regulated genes in PXO99^{A} versus PΔ*minC* and 8-24. DEGs were selected with the standards (log_2Fold Change > 1). The overlapping DEGs were represented as the number. (B) GO analysis of DEGs in the *minC* mutant 8-24. The abscissa axis represents the GO category, and the ordinate axis represents the value of significant ($P < 0.05$). (C) KEGG analysis of DEGs in the *minC* mutant 8-24. The abscissa axis represents the KEGG pathway, and the ordinate axis represents the value of significant ($P < 0.05$).
