# Supplementary material for: The MinCDE Cell Division System Participates in the Regulation of Type III Secretion System (T3SS) Genes, Bacterial Virulence, and Motility in Xanthomonas oryzae pv. oryzae
Source: Microorganisms. 2022 Jul 31;10(8):1549. doi: 10.3390/microorganisms10081549 (PMC9414521; doi:10.3390/microorganisms10081549)
Supplement: Supplementary file 1 [file microorganisms-10-01549-s001.zip › Supporting Table S1 and Table S2.pdf]

Supplementary Table S1. Bacterial strains and plasmids used in this study

**Strains or plasmids**	Relevant characteristics[a]	Source
**Strains**		
Xanthomonas. oryzae* pv. *oryzae		
PXO99^A	The *Xoo* wild-type strain, Philippine race 6	[32]
8-24	*minC* Tn5 insertion mutant of PXO99^A; Km^r	This study
24-46	*minD* Tn5 insertion mutant of PXO99^A; Km^r	This study
PΔ*minC*	*minC* deletion mutant of PXO99^A	This study
PΔ*minD*	*minD* deletion mutant of PXO99^A	This study
PΔ*minCDE*	Triple *minC, minD* and *minE* deletion mutant of PXO99^A	This study
C8-24	8-24 harbouring pML123-*minC*; Km^r, Gm^r	This study
CPΔ*minC*	PΔ*minC* harbouring pML123-*minC*; Gm^r	This study
CPΔ*minD*	PΔ*minD* harbouring pML123-*minD*; Gm^r	This study
CPΔ*minCDE*	PΔ*minCDE* harbouring pML123-*minCDE*; Gm^r	This study
PΔ*hrpG*	*hrpG* deletion mutant of PXO99^A	This laboratory
PΔ*hrcU*	*hrcU* deletion mutant of PXO99^A	This laboratory
PΔ*trh*	*trh* insertion mutant of PXO99^A; Km^r	This laboratory
PΔ*xrvA*	*xrvA* insertion mutant of PXO99^A; Km^r	This laboratory
PΔ*zur*	*zur* insertion mutant of PXO99^A; Km^r	This laboratory
PΔ*rpfC*	*rpfC* insertion mutant of PXO99^A; Km^r	This laboratory
PΔ*rpfG*	*rpfG* insertion mutant of PXO99^A; Km^r	This laboratory
PΔ*rpfF*	*rpfF* insertion mutant of PXO99^A; Km^r	This laboratory
PΔ*clp*	*clp* insertion mutant of PXO99^A; Km^r	This laboratory
**Escherichia coli**		
DH5a	φ901ac ZΔM15, recA1	Invitrogen
**Plasmids**		
pHG2-*hrpF*	The *Xoo hrpX* promoter cloned in pHG2; Sp^r	[33]
pHG3-*hrpB1*	The *Xoo hrpB1* promoter cloned in pHG3; Sp^r	[36]
pHG2-*hrpG*	The *Xoo hrpG* promoter cloned in pHG2; Sp^r	[36]
pHG2-*hrpX*	The *Xoo hrpX* promoter cloned in pHG2; Sp^r	[36]
pH1-*hrpG*::FLAG	The fusion of *hrpG*::FLAG cloned in pH1; Sp^r	[33]
pH3-*hrpX*::FLAG	The fusion of *hrpX*::FLAG cloned in pH3; Sp^r	[33]
pH3-*hrpB1*::FLAG	The fusion of *hrpB1*::FLAG cloned in pH3; Sp^r	[33]
pML123-*minC*	The *minC* complementary fragment cloned in pML123; Gm^r	This study
pML123-*minD*	The *minD* complementary fragment cloned in pML123; Gm^r	This study
pML123-*minCDE*	The *minCDE* complementary fragment cloned in pML123; Gm^r	This study
pHM1-*gfp*	A high-copy vector harbouring a *gfp* gene derived from pHM1; Sp^r, Ap^r	This laboratory
pHG2-*hrpG-post*	The post transcriptional fusion of *hrpG*::*uidA* cloned in pH2; Sp^r	This laboratory

[a]Ap^r, ampicillin resistance; Km^r, kanamycin resistance; Gm^r, gentamycin resistance; Sp^r,

streptomycin resistance

Supplementary Table S2: Primer sequences used in this study

Primers Name	Primer Sequences (5'-3')	Description
minC up-F	GTGTCTAGACGCATGTAATAATCCAACGC	
minC up-R	ATGAAGCTTAGCACACCGGCTTCGTGCAG	For *minC* deletion mutant construction
minC down-F	GAGAAGCTTATCTTCTGCCGCGACTTCCA	
minC down-R	ACTCCCGGGCAGCCAGGTCCTTGAGCACC	
minD up-F	ACTCTGCAGCGATGCGGCCTTGCTGTTC	
minD up-R	GACTCTAGAGGCTTGCGCTGGTGGTGGT	For *minD* deletion mutant construction
minD down-F	AAGTCTAGAGCGTGGAAGGCGGCGAGATG	
minD down-R	AGAGGATCCCGGTCGGGTCACAGGCAACA	
minCDE up-F	ACACTGCAGTTATATGCCGATGTGCAGA	
minCDE up-R	GACTCTAGAGGTCACGCGCTCACGCATT	For *minC*, *minD* and *minE* triple deletion mutant construction
minCDE down-F	AAGTCTAGAGAAGACGGCGACAAGTAAG	
minCDE down-R	AGAGGATCCAGTGCCTCGGTCGAACAGT	
GNAT-*minC*-F	CGGGTTTCTGGTCGGATTCG	For comfirmation of GNAT-*minC* operon
GNAT-*minC*-R	GGAAGTCGCGGCAGAAGATG	
minC-D-F	ACCACCAGCGCAAGCCTGGC	For comfirmation of *minC*-D operon
minC-D-R	GTTCGCCATCCTTGACCAGA	
minD-E-F	ACCACCAGCGCAAGCCTGGC	For comfirmation of *minD*-E operon
minD-E-R	GTTCGCCATCCTTGACCAGATC	
minE-PXO_04466-F	ATGGGCCTGCTCGATTTTCT	For comfirmation of *minE-PXO_04466* operon
minE- PXO_04466-R	TCACGCAGGCAACAAGCCGC	
minC com-F	GAGAAGCTTGAACAACAATGCCGGACTG	For *minC* complementray fragment
minC com-R	GATGTCGACACCACGCGCCGCTCGCAGC	
minCDE com-F	GAGAAGCTTGAACAACAATGCCGGACTG	For *minC*, *minD* and *minE* complementray fragment
minCDE com-R	GATGTCGACTCGCTCGCGGTCGGGTCACAG	
minD com-F	GAGAAGCTTATCGATGTTGACGTACTTC	For *minD* complementray fragment
minD com-R	GATGTCGACAGTTGTATGCGGAGAACTG	
hrpB1-F	TTCGATGCATGGATTTCGATCAAGC	*hrpB1* gene fragment for qRT-PCR
hrpB1-R	CGCCGGTGCGGACGTTGGGGTAGTT	
rpfC-F	ACCACGCGTCTGGATGTAGAGCAGAAGG	*rpfC* gene fragment for qRT-PCR
rpfC-R	GCACCTCTTCCACCAGCGACAGCAGACT	
rpfG-F	TGGAGCAGCGCTTGCTGGCCAGCATGAA	*rpfG* gene fragment for qRT-PCR
rpfG-R	CGACAACCCCAGTTGCTCGGCAATCAGG	
rpfF-F	CAGTTGCTTGGCATGGGTCT	*rpfF* gene fragment for qRT-PCR
rpfF-R	TGGTACTGCGGTGGTCATTT	
clp-F	GTTGTTCATCGAATCCGATACCCGCGAG	*clp* gene fragment for qRT-PCR
clp-R	ATCTTCGGCGCATCCGGCGACAGGCTGG	
xrvA-F	CGAACTGCAGAAGCTGGAAGAACAGGAG	*xrvA* gene fragment for qRT-PCR
xrvA-R	CTTCGCTGCGGCCGCTCTTGACCACGTT	
trh-F	CAACTTGAAGCCGAAGGCCGCATCTACC	*trh* gene fragment for qRT-PCR

trh-R	ACGTACTGCATGAAACCGGCGATGCTGG	
rpoD qRT-F	CGACAACACCACCAACATCAATC	*rpoD* gene fragment for qRT-PCR
rpoD qRT-R	GCTTACCGACCTCTTCCAACG	
gyrB qRT-F	CGGCACTTACGACTCCAGCAAG	*gyrB* gene fragment for qRT-PCR
gyrB qRT-R	CGACCAGGATTTTCACCACGATG	

Note: Underlined bases indicate restriction enzyme sites
